# Prevalence of Extended-Spectrum β-Lactamase-Producing *Escherichia coli*, *Klebsiella pneumoniae* and *Enterobacter cloacae* in Wastewater Effluent in Blantyre, Malawi

**DOI:** 10.3390/antibiotics14060562

**Published:** 2025-05-30

**Authors:** Edna Ibrahim, Charity Mkwanda, Edward Masoambeta, Luigia Scudeller, Tomislav Kostyanev, Hussein H. Twabi, Yohane K. Diness, Jobiba Chinkhumba, Janelisa Musaya, Rajhab S. Mkakosya, Surbhi Malhotra-Kumar, Chantal M. Morel, Save Kumwenda, Chisomo L. Msefula

**Affiliations:** 1 Pathology Department, Kamuzu University of Health Sciences, Blantyre 312200, Malawi; 202240140007@kuhes.ac.mw (C.M.); 202250010008@kuhes.ac.mw (E.M.); htwabi@kuhes.ac.mw (H.H.T.); jchinkhumba@kuhes.ac.mw (J.C.); jmusaya@kuhes.ac.mw (J.M.); rmkakosya@kuhes.ac.mw (R.S.M.); 2 School of Science and Technology, Malawi University of Business and Applied Sciences, Blantyre 312200, Malawi; skumwenda@mubas.ac.mw; 3 Scientific Direction, Fondazione IRCCS Policlinico San Matteo, Viale Camillo Golgi 19, 27100 Pavia, Italy; l.scudeller@smatteo.pv.it; 4 Laboratory of Medical Microbiology, Vaccine and Infectious Disease Institute, University of Antwerp, Antwerp, Universiteitsplein 1, 2610 Wilrijk, Belgium; tomislav.simeonov.kostyanev@rsyd.dk (T.K.); surbhi.malhotra@uantwerpen.be (S.M.-K.); 5 Institute of Life Course and Medical Sciences, University of Liverpool, Liverpool L7 8TX, UK; 6 Malawi Liverpool Wellcome Program, Blantyre 312200, Malawi; ydiness@mlw.mw; 7 Institute for Public Health and Hygiene, University Hospital Bonn, Venusberg-Campus 1, 53127 Bonn, Germany; chantal.morel@unibe.ch; 8 KPM Center for Public Management, University of Bern, Schanzeneckstrasse 1, 3012 Bern, Germany; 9 Multidisciplinary Center for Infectious Diseases, University of Bern, Hallerstrasse 6, 3012 Bern, Germany

**Keywords:** antimicrobial resistance, Enterobacteriaceae, extended spectrum β-lactamases, sewage effluent, wastewater treatment plants

## Abstract

**Background/Objectives**: Wastewater treatment plants (WWTPs) serve as a sink for both antimicrobial residues and bacteria carrying resistant genes, which are later disseminated into the environment, facilitating the spread of antimicrobial resistance. This study investigated the presence of extended-spectrum beta-lactamase (ESBL) producing *Escherichia coli (Ec)*, *Klebsiella pneumoniae (Kp)*, and *Enterobacter cloacae (Enc)* in effluent from WWTP in Blantyre, Malawi, to generate evidence and provide baseline information for interventions. **Methods**: Selective chromogenic agar was used to identify ESBL-producing bacteria. **Results**: A total of 288 samples were collected between April 2023 and March 2024, and 97.6% (281/288) yielded one or more presumptive ESBL isolates. Bacterial growth was confirmed as 48.9% *Ec* (255/522), 33.0% *Kp* (172/522), and 10.0% *Enc* (52/522). Antibiotic susceptibility testing showed the highest resistance to ceftriaxone (*Ec*, 100.0%; *Kp*, 98.3%; *Enc*, 100.0%) and the lowest resistance to meropenem (*Ec*, 6.3%, *Kp*, 1.2%; *Enc*, 3.8%) among the antibiotics that were tested. Multiple antibiotic resistance phenotypes were observed in 73.1% of the isolates, with the most prevalent phenotype being amoxicillin + clavulanate/cotrimoxazole/doxycycline/ciprofloxacin/gentamicin/azithromycin/ceftriaxone (55, 15.7%). **Conclusions**: The study demonstrated ongoing environmental contamination with antibiotic-resistant bacteria from sewage effluent. Therefore, the functionality of WWTPs should be improved to minimize the release of these organisms into the environment.

## 1. Introduction

The environment is a receiver of all waste of human or animal origin in the form of feces, urine, and pharmaceutical or sewage effluent that may carry with it selected resistant bacteria and antibiotic residues into soil, water, or air [1,2,3,4,5]. Wastewater treatment plants (WWTPs) are considered a major source of bacteria carrying resistant genes into the environment [6]. Although it is well known that wastewater treatment systems are not designed to remove chemical pollutants and do not completely remove the pathogenic bacteria, it is essential to determine the levels of discharge to the environment. Thus, studying environmental contamination levels from WWTP will lead to a deeper understanding of how antibiotic-resistant infections emerge and how to regulate the use of treated wastewater. Bacteria in the environment and contact with effluent wastewater are exposed to antimicrobials in sub-optimal or repeated exposures that provide a platform for resistance development [4,7,8]. In addition, pathogenic antibiotic-resistant bacteria contaminating the environment from wastewater treatment plants may interact with other environmental microbiota and transmit resistance and virulence genes [7]. Resistant bacterial species in the environment may transmit to humans and also to domesticated and wild animals, causing incurable diseases, thereby amplifying the antimicrobial resistance (AMR) problem further [9].

Antibiotic resistance is a growing concern worldwide and continually emerging due to many contributing factors that differ from place to place. Microorganisms perpetually mutate and evolve, making it difficult to treat diseases, thereby affecting most of the gains achieved in the management of infectious diseases [10]. In 2019, about 1.27 million deaths were attributable to AMR bacteria in sub-Saharan Africa [11]. In Malawi, a 20-year study at Queen Elizabeth Central Hospital (QECH) between 1998 and 2017 reported an increase in AMR associated with bloodstream infections, a major cause of death in patients [12]. A more recent study conducted at the same hospital in Malawi revealed that patients with bloodstream infections caused by bacteria resistant to third-generation cephalosporins have longer hospital stays and are linked to higher fatality rates [13]. The persistence of AMR is attributed to the increased use of antimicrobials in the human [14] and animal sectors [15] and also the release of effluent from wastewater treatment systems, animal farms, and pharmaceutical industries into the environment that may select for antibiotic resistance [1,2,3]. In order to help in the development of preventative interventions, extensive and comprehensive data on antimicrobial-resistant pathogens, their effect on human and animal health, and the associated consequences are being generated [12,16,17]. Relatively little is known about AMR in the environment, which is a more expansive and varied domain. Therefore, there is a need for environmental surveillance to generate evidence for resistant microbes in the environmental sector and also to define emergence and transmission pathways. This study investigated the presence of ESBL-producing pathogens in effluent from WWTPs in Blantyre, Malawi, to complement the data already being collected in the human and animal sectors.

## 2. Results

### 2.1. Isolation and Identification of ESBL Organisms in WWTPs

A total of 288 effluent samples were collected between 3rd April 2023 and 27th March 2024 and 281/288 samples (97.57%) yielded one or more presumptive ESBL isolates. Of these, 7 contained ESBL *E. coli* only (2.5%), 16 samples contained ESBL *Enterobacter cloacae* only (5.7%), 36 samples contained ESBL *E. coli* and ESBL *Enterobacter cloacae* (12.8%), 172 contained ESBL *E. coli* and ESBL *Enterobacter cloacae* (61.2%), and 41 samples contained ESBL *E. coli* and another confirmed organisms or organisms with low discrimination profiles (14.6%). The remaining eight samples contained other ESBL growth that was neither pink nor blue (2.8%); these colonies were not processed further, as shown in Table 1.

### 2.2. Resistance Profiles of Isolated ESBL-Producing E. coli, K. pneumoniae, and Enterobacter Cloacae

**Figure 1:**
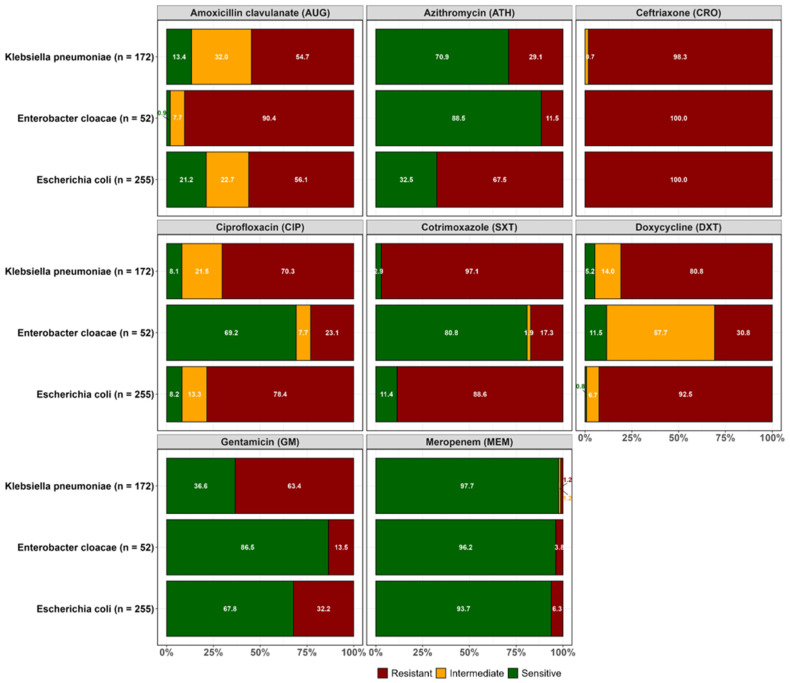
Overall antimicrobial resistance patterns for confirmed ESBL-positive organisms cultured from the effluent collected in two wastewater treatment plants.

Overall, resistance to the third-generation cephalosporin ceftriaxone was common in the three ESBL-producing pathogens, as expected. Considering each species individually, ESBL *E. coli* isolates showed the highest resistance to ceftriaxone (100.0%), followed by doxycycline (92.5%), cotrimoxazole (88.6%), ciprofloxacin (78.4%), and gentamicin (32.2%). Relatively, the lowest resistance rate was observed to meropenem in ESBL *E. coli* (6.3%). For ESBL *Klebsiella pneumoniae*, the highest resistance rate was observed for ceftriaxone (98.3%), followed by doxycycline (80.8%), cotrimoxazole (97.1%), and ciprofloxacin (70.3%). However, lower resistance was observed for azithromycin (29.1%) and the lowest resistance for meropenem (1.2%). ESBL *Enterobacter cloacae* were more susceptible to most of the antibiotics that were tested, except for ceftriaxone (100%), a third-generation cephalosporin, and amoxicillin clavulanate (90.4%), to which intrinsic resistance has been reported (Figure 1).

**Figure 2:**
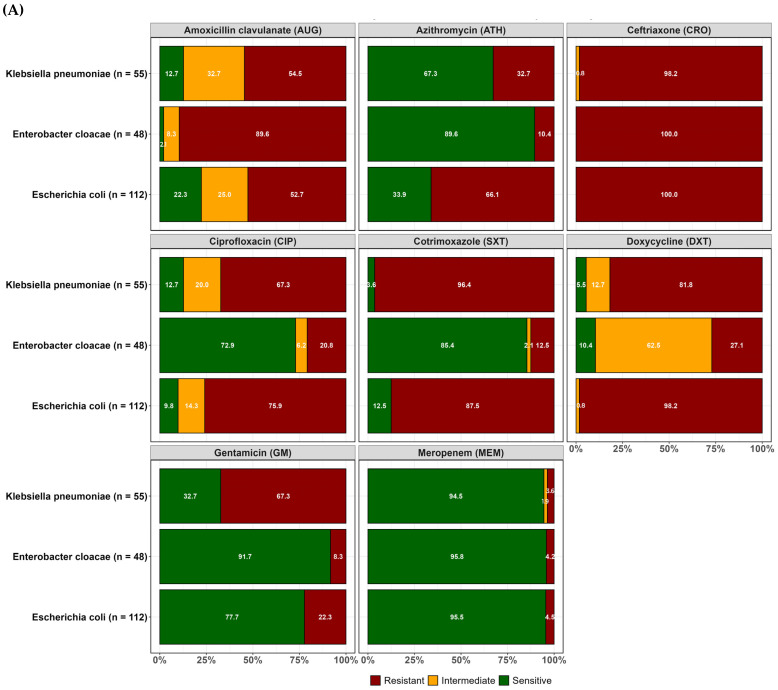

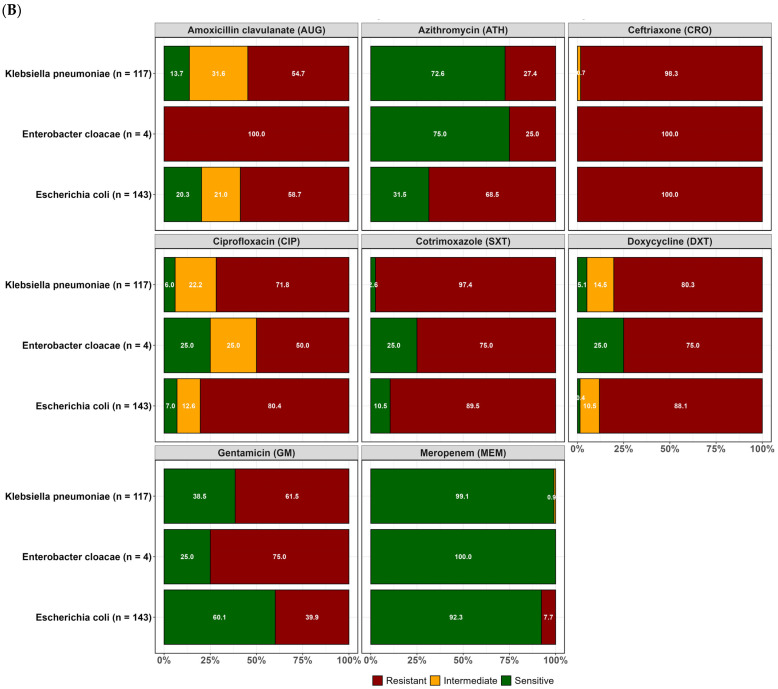
Antimicrobial resistance patterns for organisms cultured from (**A**) the Blantyre treatment plant and (**B**) the Soche Sewage Treatment Plant.

Resistance to at least one of the antibiotics tested was observed in the confirmed ESBL-positive isolates recovered from both WWTPs, as highlighted in Figure 2. The results suggest a lack of significant differences between the two WWTPs in the levels of resistance to most of the antibiotics tested, as presented in Tables S1–S3, except for a few antibiotics against *E. coli* (DXT and GM) and against *E. cloacae* (DXT and SXT), where the levels of resistance were significantly different (*p* < 0.05).

### 2.3. Prevalence of Multiple Antibiotic-Resistance Phenotype and Multiple Antibiotic Resistance Index ESBL E. coli, ESBL K. pneumoniae and ESBL E. cloacae

A total of 350 (73.1%) isolates were resistant to three or more classes of antibiotics. Among the multiple resistant isolates that were observed, 51.4% (180/350) were ESBL *E. coli*, 43.1% (151/350) were ESBL *K. pneumoniae,* and 5.4% (19/350) were ESBL *E. cloacae*. The most prevalent multiple antibiotic-resistant phenotypes (MARP) among the isolates were AUG-SXT-DXT-CIP-GM-ATH-CRO (55, 15.7%). Six ESBL *E. coli* isolates were resistant to all eight antibiotics that were tested. The multiple antibiotic resistance index (MARI) ranged from 0.4 to 1 with 29.1% of the MAR phenotype isolates having a MARI of 0.6 (Table 2). The average MARI at the two sites was comparable with Blantyre at 0.61 and Soche at 0.58.

### 2.4. Effect of Temperature and Rainfall on the Prevalence of Resistant ESBL Organisms

**Figure 3:**
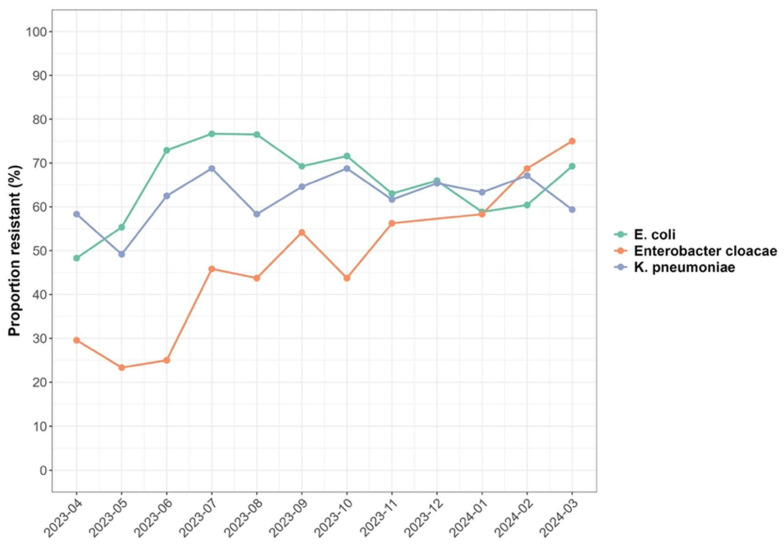
Trends in the proportion of all the ESBL organisms resistant to antibiotics isolated each month over 12 months.

The analysis showed present and ongoing environmental contamination with multiple antibiotic-resistant ESBL bacteria across the 12 months of investigation (Figure 3). It was observed that increases in minimum temperature were associated with an increase in the prevalence of resistant ESBL organisms with an IRR of 1.04 (95% CI: 1.01; 1.08). Across the sampled locations, an increase in minimum temperature was associated with an increase in the prevalence of resistant ESBL organisms isolated by 1.41(95% CI: 1.30; 1.53) (*p* < 0.001) for Soche WWTP. Results also showed a decrease in prevalence of resistant ESBL *K. pneumoniae* and ESBL *Enterobacter cloacae* with an IRR of 0.75 (95% CI: 0.23; 0.32) and 0.27 (95% CI: 0.69, 0.82), respectively, with increasing minimum temperature. Conversely, an increase in minimum temperature was associated with increasing antibiotic resistance to CRO, SXT, DXT, and CIP with an IRR of 1.62 (95% CI: 1.40, 1.88), 1.58 (95% CI: 1.35, 1.84), 1.40 (95% CI: 1.20, 1.63), and 1.33 (95% CI: 1.40, 1.56), respectively.

Neither maximum temperature nor rainfall had any effect on the prevalence of resistant ESBL organisms, as summarized in Table 3.

## 3. Discussion

This study has shown environmental contamination with ESBL-producing *E. coli*, *K. pneumoniae,* and *Enterobacter cloacae* from sewage effluent from two WWTPs in Blantyre, Malawi. A majority of the isolates were resistant to ceftriaxone, as expected, but there are still high levels of susceptibility among the isolates to meropenem. Additionally, the different ESBL-producing isolates presented multiple antibiotic resistance phenotypes with MARI ranging from 0.4 to 1, suggesting high antibiotic pressure or use.

The presence of organisms in the wastewater effluent in Blantyre, Malawi mirrors the observations across the globe reporting ESBL-producing Enterobacterales in effluent wastewater in South Africa [18], Hungary [19], The Netherlands [20], Tokyo [21], and Algeria [22]. This suggests the need for continuous surveillance for the levels of resistant pathogens in the environment and the improved functionality of wastewater treatment plants across the socioeconomic spectrum, encompassing high and also low- and middle-income countries.

Malawi is a low-income country with poor sanitation facilities such as pit latrines, sewage systems, and WWTPs. Solid and fecal waste are not always disposed of appropriately and in designated places [23]. This study persistently found resistant ESBL-producing organisms in effluent from WWTPs over a one-year period, which is a concern because the effluent passes through gardens before entering a river. Of note, there are frequent reports of multidrug-resistant bloodstream infections of *E. coli* and *K. pneumoniae* from a large tertiary hospital located within the catchment area of the Soche WWTP, included in this study [12,13]. An investigation into the surrounding communities’ exposure to the effluent and the ESBL-producing organisms is required to design appropriate infection prevention and control measures.

A wide variety of antibiotic resistance profiles were found among the ESBL-producing *E. coli*, *K. pneumoniae*, and *E. cloacae* isolates characterized in this study. The most commonly detected multidrug resistance profile (AUG-SXT-DXT-CIP-GM-ATH-CRO) among these environmental isolates included resistance to widely consumed antibiotics, nationally, in the human sector in Malawi [24]. There is a potential that the levels of antibiotic consumption are contributing to the emergence of antibiotic resistance [14]. The calculated multiple antibiotic resistance index in all three ESBL bacterial species in this study is greater than 0.2 predictive of drug resistance pollution in the environment or high use of antibiotics [6,25,26,27]. Furthermore, the characterized antibiotic resistance phenotypes in this study are similar to those being reported in isolates from human samples [28,29]. The potential for horizontal transfer of resistance genes between human and environmental isolates is possible in this setting. Genome-level assessments performed in other settings have determined similarity in ESBL-producing isolates from the environmental waste waters and those isolated from human infection [30]. Genomic surveillance of these MDR pathogens from wastewater and other external environments is needed to ascertain the movement of the bacteria between the environment and the host being infected, whether human or animal.

High levels of resistance to antibiotics in the access (AUG, DXT, GM, and SXT) and watch (ATH, CIP, CRO, and MEM) WHO categories were prevalent among the environmental isolates investigated in this study. Resistance to meropenem, which is categorized as a reserve antibiotic in Malawi, was also determined in a few of the isolates from this study. There are limited options for treating these resistant bacteria contaminating gardens and rivers in Blantyre. Carbapenems are scarcely available in public hospitals [14]. Unregulated antibiotic use at the household level in Malawi is largely limited to the access group of antibiotics, which are readily available and affordable [27]. The landscape of resistance in the environmental setting mirrors the reports of high-level resistance to access antibiotics and the low levels of carbapenem resistance in the human sector in Malawi [26,28]. The similarity in antibiotic resistance levels indicates that environmental isolates and surveillance thereof can be useful in predicting antimicrobial resistance patterns in human infection. As the country invests in infection prevention measures and also antimicrobial stewardship practices in hospitals [31], waste management regulations should also be strengthened to control the spread of ESBL-producing and carbapenem-resistant infections.

The ESBL-producing bacteria were isolated throughout a twelve-month period that included both hot and cold seasons as well as dry and wet seasons. Temperature and rainfall have been known to affect bacterial growth and persistence of bacteria in wastewater systems [32]. This study revealed an increase in the prevalence of resistant ESBL-producing bacteria with increasing minimum temperature (IRR = 1.04). Similar findings have been reported in the United States [33] substantiating that minimum temperature is associated with the increasing presence of resistant bacterial species in the environment. In terms of resistance to specific antibiotics, there was an increase in antibiotic resistance to CRO, SXT, DXT, and CIP with increasing minimum temperature. CRO, SXT, DXT, and CIP are the most commonly used antibiotics [24] which would increase the selective pressure, and also the conditions are favorable for increasing multiple antibiotic resistance. The study in the United States [33] reported similar results with increasing fluoroquinolones resistance in methicillin-resistant *S. aureus,* suggesting that minimum temperature has a role to play in AMR.

The main strength of this study was that it allowed for AMR surveillance in the environmental sector, complementing surveillance efforts in the animal and human sectors in Malawi. However, there is a need in future studies to also investigate the prevalence of non-ESBL-producing AMR pathogens in the effluent and also explore how the organisms identified in the effluent disseminate and are transmitted in the communities surrounding the wastewater treatment plants. Additionally, an assessment of the influent coming from the different and varied sources may be necessary for targeted sectoral interventions. Future studies should consider molecular methods such as polymerase chain reaction and whole genome sequencing to characterize the resistance genes and also assess the potential for horizontal gene transfer, providing insights into the spread of antibiotic resistance in these settings. It may also be necessary in follow-up studies to consider assessments of antibiotic residues in the effluent, to take into account factors selecting for resistant organisms.

In conclusion, the study focused on isolating ESBL-producing *E. coli*, *Klebsiella pneumoniae* and *Enterobacter cloacae* and determining the varied antibiotic susceptibility profiles of the identified bacteria from two WWTPs in Blantyre city. High prevalence of ESBL-producing and multiple antibiotic-resistant Enterobacterales was observed in both WWTPs. Therefore, improved and efficient wastewater management systems should be put in place to reduce the levels of AMR organisms in the environment. The study also highlights the need for improved antimicrobial stewardship programs and interventions to equally invest in environmental surveillance to fill the knowledge gaps necessary to avert the spread of AMR in Malawi.

## 4. Materials and Methods

### 4.1. Study Sites

**Figure 4:**
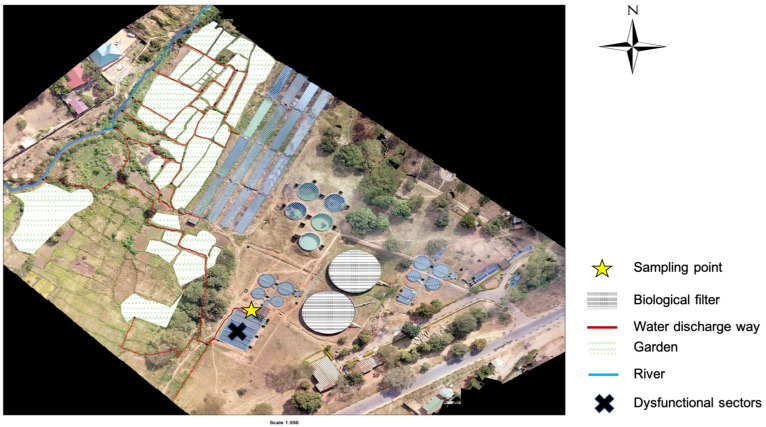
Layout of the Soche wastewater treatment plant showing the sampling point for effluent and the discharge way into nearby gardens and the river.

**Figure 5:**
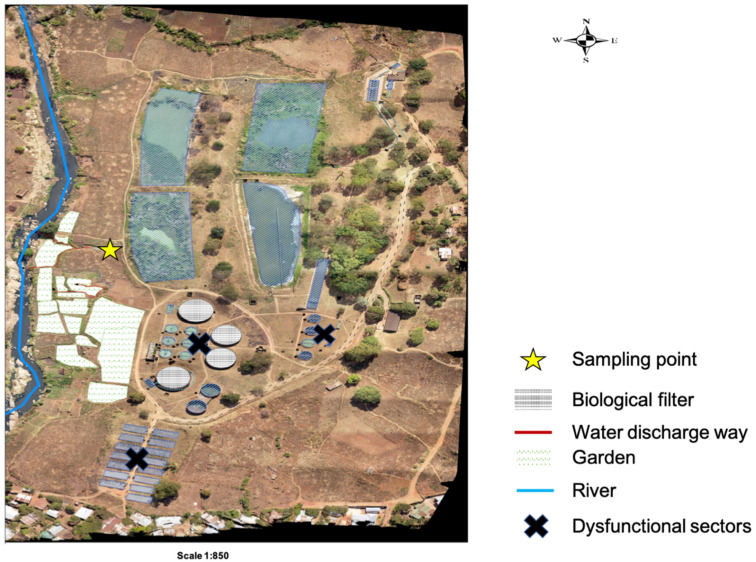
Layout of the Blantyre wastewater treatment plant showing the sampling point for effluent and the discharge way into nearby gardens and the river.

The study was conducted within Blantyre, focusing on Soche (S 15°49′13.71″ E 35°0′26.44″) and Blantyre (S 15°48′32.87″ E 34°59′20.92″) wastewater treatment plants. These two sites are of the conventional design; however, Blantyre WWTP uses stabilization ponds due to system blockage. Both treatment plants receive waste effluent from surrounding industries and households. Additionally, the Soche treatment system also receives sewage from septic tanks that are delivered by road tankers, and also sewage from Queen Elizabeth Central Hospital (QECH) [34]. QECH is a large tertiary hospital that caters to a population of about 1.3 million from Blantyre City, and also referrals from the surrounding districts [35]. The treatment plants discharge their effluent into streams, and also directly into farms that are a few kilometers after the discharging point (Figure 4 and Figure 5).

### 4.2. Sample Collection

This study collected effluent wastewater samples at the point of discharge weekly on three consecutive days: Mondays, Tuesdays, and Wednesdays from 3 April 2023 to 27 March 2024. From both sites, a 500-mL grab sample was collected every hour from 9:00 a.m. to 1:00 p.m. in sterile bottles. The samples were stored in a cooler box packed with ice blocks and were immediately transported to the laboratory for processing.

### 4.3. Sample Processing, Culture, and Bacterial Identification

Sample processing for resistant bacteria and bacterial identification was performed according to Moges et al. [7] and Teshome et al. [36]. Briefly, 200 mL aliquots from individual grab samples for each day were pooled together and mixed before analysis to make a one-liter composite sample, respectively, for each study site. A total of 500 mL of the pooled and mixed samples was filtered through a 0.45 µm pore membrane (Cellulose Nitrate Filter, Goettingen, Germany) to concentrate the bacteria. After filtration, the nitrocellulose filters were incubated in buffered peptone water (BPW) enrichment media at 37 °C overnight. After 18–22 h, the cultures were sub-cultured onto ESBL chromogenic agar (CHROMagar^TM^, La Plaine Saint-Denis, France) with ESBL supplement to check for ESBL-producing bacteria. On the third day, the plates were characterized for pink colonies indicative of *E. coli* growth, and blue colonies indicative of either *K*. *pneumoniae* or *E. cloacae* growth. One pink colony of presumptive *E. coli* and one blue colony (presumptive *K. pneumoniae* or *E. cloacae*) from each plate were then isolated and further subcultured onto fresh ESBL CHROMagar for 18–22 h at 37 °C to make a pure culture. The pure culture was then subcultured onto nutrient agar, followed by colony growth identification using the analytical profile index (API) 20E Kit (Biomerieux).

### 4.4. Antimicrobial Susceptibility Testing

The disk diffusion method was used to measure antibiotic susceptibility of the isolates according to Moges et al. [7] and Teshome et al. [36]. A pure colony from nutrient agar was emulsified in sterile 4–5 mL phosphate-buffered saline and homogeneously spread on Mueller–Hinton agar plates. This was followed by dispersing the antibiotic disk of azithromycin (15 μg), amoxicillin–clavulanate (30 μg), ciprofloxacin (5 μg), ceftriaxone (30 μg), cotrimoxazole (1.25/23.75 μg), doxycycline (30 μg), gentamicin (10 μg), and meropenem (10 μg) onto the plates and incubating aerobically at 37 °C for 18–24 h. After incubation, results were read and interpreted by referring to the Clinical Laboratory Standard guidelines (CLSI) [37].

### 4.5. Multiple Antibiotic-Resistant Phenotype and Multiple Antibiotic Resistance Index

Multiple antibiotic resistance (MAR) was considered for isolates that were resistant to three or more classes of antibiotics [6]. Precisely, a/b, where a represents the number of antibiotic isolates that were resistant and b, the number of antibiotics tested [38]. Whereas the multiple antibiotic resistance index (MARI) was calculated as a proportion of antibiotics that an isolate was resistant to, compared to the total number of antibiotics that were tested [6,25].

### 4.6. Temperature and Rainfall Data

The temperature and rainfall data used in this study were requested from the Department of climate change and meteorological services in Malawi. Chichiri data were used for BTP WTP, and Mpemba data were used for Soche WTP.

### 4.7. Statistical Analysis

All data collected from the laboratory were organized and quality checked using Microsoft Excel. Statistical analysis was performed in R version 4.4.0, where figures and tables were also computed. Descriptive statistics were used to summarize data on samples. Poisson regression analysis with chi-square goodness-of-fit was applied at 95% confidence interval to check for the effect of rainfall and temperature on the prevalence of resistant ESBL organisms (*p*-value < 0.05 was considered for significance). Poisson regression outputs were presented as incidence rate ratio (IRR) to define the effect or risk (where IRR = 1, no effect; IRR < 1, negative effect; IRR > 1, positive effect).

## Figures and Tables

**Table 1:** Proportion of different species of ESBL-producing organisms isolated from 281 effluent samples from two WWTPs, Blantyre and Soche.

Species of ESBL Organisms Isolated from Each Sample	Number of Samples Per Site		
	Blantyre, N = 138	Soche, N = 143	Overall, N = 281
*E. coli* only	4/138	3/143	7/281
	(2.90%)	(2.10%)	(2.50%)
*Enterobacter cloacae* only	16/138	0/143	16/281
	(11.60%)	(0.00%)	(5.70%)
*Enterobacter asberiae* only	1/138	0/143	1/281
	(0.70%)	(0.00%)	(0.40%)
*E. coli* and *Enterobacter cloacae*	32/138	4/143	36/281
	(23.20%)	(2.80%)	(12.80%)
*E. coli* and *K. pneumoniae pneumoniae*	55/138	117/143	172/281
	(39.90%)	(81.80%)	(61.20%)
*E. coli* and *K. pneumoniae ozaenae*	3/138	2/143	5/281
	(2.20%)	(1.40%)	(1.80%)
*Proteus vulgaris*	1/138	0/143	1/281
	(0.70%)	(0.00%)	(0.40%)
*E. coli* and *Rahnella aqualitis*	0/138	1/143	1/281
	(0.00%)	(0.70%)	(0.40%)
*E. coli* and *Raoultella ornithinolytica*	0/138	2/143	2/281
	0.00%	(1.40%)	(0.70%)
*E. coli* and Low discrimination profiles (*Aeromonas*, other *Klebsiella pneumoniae* subspp, *Pantoea,* and *Vibrio* species)	19/138	14/143	33/281
	(13.80%)	(9.80%)	(11.70%)
Other ESBL growth, but no pink or blue colonies	8/138	0/143	8/281
	(5.80%)	(0.00%)	(2.80%)

Pink for presumptive *E. coli*; Blue for presumptive *K. pneumoniae* or Enterobacter cloacae.

**Table 2:** Multiple antibiotic resistance (MAR) phenotypes and index among ESBL organisms.

		Soche			Blantyre			
MAR Phenotype	*E. coli* (143)	*K. pneumoniae* (117)	*E. cloacae* (4)	*E. coli* (112)	*K. pneumoniae* (55)	*E. cloacae* (48)	Total	MAR Index
AUG, MEM, SXT, DXT, CIP, GM, ATH, CRO	6	0	0	0	0	0	6	1
AUG, SXT, DXT, CIP, GM, ATH, CRO	34	6	1	11	1	2	55	0.9
AUG, SXT, DXT, GM, ATH, CRO	0	1	0	1	3	0	5	0.8
AUG, SXT, GM, ATH, CRO	0	0	0	0	1	0	1	0.6
SXT, GM, ATH, CRO	0	1	0	0	0	0	1	0.5
SXT, ATH, CRO	1	1	0	0	0	1	3	0.4
SXT, CIP, GM, CRO	0	1	0	0	0	0	1	0.5
SXT, CIP, GM, ATH, CRO	0	1	0	0	0	0	1	0.6
SXT, DXT, CIP, GM, ATH, CRO	9	5	0	5	4	0	23	0.8
SXT, CIP, ATH, CRO	1	0	0	0	0	0	1	0.5
SXT, CIP, CRO	0	2	0	0	0	0	2	0.4
SXT, DXT, CRO	3	6	0	6	0	0	15	0.4
SXT, DXT, CIP, CRO	9	7	0	7	4	1	28	0.5
AUG, SXT, DXT, CIP, CRO	4	5	0	6	3	0	18	0.6
AUG, SXT, DXT, CRO	5	2	0	2	0	1	10	0.5
AUG, SXT, CRO	1	0	0	0	0	0	1	0.4
AUG, MEM, SXT, DXT, ATH, CRO	0	0	0	1	0	0	1	0.8
AUG, SXT, DXT, CIP, GM, CRO	2	30	1	1	10	1	45	0.8
AUG, SXT, DXT, GM, CRO	0	4	1	0	3	0	8	0.6
DXT, CIP, GM, CRO	1	0	0	0	0	1	2	0.5
SXT, DXT, GM, CRO	1	5	0	0	2	0	8	0.5
SXT, DXT, CIP, GM, CRO	1	6	0	5	4	0	16	0.6
AUG, MEM, SXT, DXT, CIP, ATH, CRO	3	0	0	3	0	0	6	0.9
SXT, DXT, GM, ATH, CRO	0	1	0	1	1	0	3	0.6
AUG, SXT, CIP, ATH, CRO	0	0	0	2	0	0	2	0.6
AUG, MEM, SXT, CIP, GM, CRO	0	0	0	0	1	0	1	0.8
AUG, MEM, DXT, CIP, ATH, CRO	0	0	0	0	0	1	1	0.8
AUG, MEM, DXT, CIP, CRO	0	0	0	0	0	1	1	0.6
AUG, CIP, CRO	0	1	0	0	0	2	3	0.4
SXT, DXT, ATH, CRO	2	4	0	2	2	0	10	0.5
AUG, DXT, CRO	0	0	0	1	0	4	5	0.4
AUG, SXT, CIP, GM, CRO	1	7	0	0	3	0	11	0.6
AUG, MEM, SXT, DXT, GM, ATH, CRO	1	0	0	0	0	0	1	0.9
AUG, CIP, GM, CRO	1	0	0	0	0	0	1	0.5
AUG, MEM, CRO	1	0	0	0	0	0	1	0.4
SXT, DXT, CIP, ATH, CRO	17	7	0	13	3	0	40	0.6
DXT, CIP, ATH, CRO	1	1	0	2	1	0	5	0.5
DXT, CIP, CRO	2	0	0	2	1	0	5	0.4
DXT, GM, ATH, CRO	0	0	0	1	0	0	1	0.5
AUG, DXT, CIP, CRO	1	0	0	0	0	0	1	0.5
AUG, DXT, CIP, ATH, CRO	0	0	0	0	0	1	1	0.6

AUG, amoxicillin–clavulanate; MEM, meropenem; SXT, cotrimoxazole; DXT, doxycycline; CIP, ciprofloxacin; GM, gentamicin; ATH, azithromycin; CRO, ceftriaxone.

**Table 3:** Effect of temperature and rainfall on resistant ESBL organisms over 12 months.

	Univariable Poisson Model			Multivariable Poisson Model		
Characteristic	IRR ^1^	95% CI ^1^	*p*-Value	IRR ^1^	95% CI ^1^	*p*-Value
Minimum Temperature	1.02	1.00, 1.04	0.080	1.04	1.01, 1.08	0.019
Maximum Temperature	1.00	0.98, 1.02	0.821	1.00	0.97, 1.02	0.637
Total rainfall in Chichiri	1.00	1.00, 1.00	0.015	1.00	1.00, 1.00	0.561
Total rainfall in Mpemba	1.00	1.00, 1.00	0.133	1.00	1.00, 1.00	0.564
**Wastewater treatment plant**			<0.001			<0.001
Blantyre	—	—		—	—	
Soche	1.41	1.30, 1.53		1.33	1.22, 1.46	
**Organism**			<0.001			<0.001
*E. coli*	—	—		—	—	
*Enterobacter cloacae*	0.27	0.23, 0.32		0.30	0.25, 0.35	
*K. pneumoniae*	0.75	0.69, 0.82		0.71	0.65, 0.78	
**Antibiotic**			<0.001			<0.001
Amoxicillin clavulanate (AUG)	—	—		—	—	
Azithromycin (ATH)	1.01	0.85, 1.20		0.95	0.79, 1.14	
Ceftriaxone (CRO)	1.62	1.40, 1.88		1.74	1.48, 2.04	
Ciprofloxacin (CIP)	1.33	1.14, 1.56		1.33	1.13, 1.58	
Cotrimoxazole (SXT)	1.58	1.35, 1.84		1.52	1.29, 1.79	
Doxycycline (DXT)	1.40	1.20, 1.63		1.47	1.25, 1.73	
Gentamicin (GM)	0.86	0.71, 1.03		0.86	0.71, 1.04	
Meropenem (MEM)	0.35	0.21, 0.53		0.31	0.19, 0.48	

^1^ IRR = incidence rate ratio, CI = confidence interval.

## Data Availability

The data presented in this study are available from the corresponding authors upon reasonable request.

## Supplementary Materials

The following supporting information can be downloaded at: https://www.mdpi.com/article/10.3390/antibiotics14060562/s1, Table S1: Antibiotic susceptibility results of *E. coli* isolates per sampling area; Table S2: Antibiotic susceptibility results of *K. pneumoniae* isolates per sampling area; Table S3: Antibiotic susceptibility results of *E. cloacae* isolates per sampling area.
