# Supplementary material for: Prevalence of Extended-Spectrum β-Lactamase-Producing Escherichia coli, Klebsiella pneumoniae and Enterobacter cloacae in Wastewater Effluent in Blantyre, Malawi
Source: Antibiotics (Basel). 2025 May 30;14(6):562. doi: 10.3390/antibiotics14060562 (PMC12189357; doi:10.3390/antibiotics14060562)
Supplement: Supplementary file 1 [file antibiotics-14-00562-s001.zip › antibiotics-3575569-supplementary.pdf]

Table S1: Antibiotic susceptibility results of *E. coli* isolates per sampling area

**Characteristic**	**Total *E. coli* isolates*1***	**Sewage Location**		**p-value2**
		**Blantyre**, N = 112*1*	**Soche**, N = 143*1*	
**Amoxicillin clavulanate (AUG)**				0.6
**I**	58/255 (22.7%)	28/112 (25.0%)	30/143 (21.0%)	
**R**	143/255 (56.1%)	59/112 (52.7%)	84/143 (58.7%)	
**S**	54/255 (21.2%)	25/112 (22.3%)	29/143 (20.3%)	
**Meropenem (MEM)**				0.3
**R**	16/255 (6.3%)	5/112 (4.5%)	11/143 (7.7%)	
**S**	239/255 (93.7%)	107/112 (95.5%)	132/143 (92.3%)	
**Cotrimoxazole (SXT)**				0.6
**R**	226/255 (88.6%)	98/112 (87.5%)	128/143 (89.5%)	
**S**	29/255 (11.4%)	14/112 (12.5%)	15/143 (10.5%)	
**Doxycycline (DXT)**				0.004
**I**	17/255 (6.7%)	2/112 (1.8%)	15/143 (10.5%)	
**R**	236/255 (92.5%)	110/112 (98.2%)	126/143 (88.1%)	
**S**	2/255 (0.8%)	0/112 (0.0%)	2/143 (1.4%)	
**Ciprofloxacin (CIP)**				0.6
**I**	34/255 (13.3%)	16/112 (14.3%)	18/143 (12.6%)	
**R**	200/255 (78.4%)	85/112 (75.9%)	115/143 (80.4%)	
**S**	21/255 (8.2%)	11/112 (9.8%)	10/143 (7.0%)	
**Gentamicin (GM)**				0.003
**R**	82/255 (32.2%)	25/112 (22.3%)	57/143 (39.9%)	
**S**	173/255 (67.8%)	87/112 (77.7%)	86/143 (60.1%)	
**Azithromycin (ATH)**				0.7
**R**	172/255 (67.5%)	74/112 (66.1%)	98/143 (68.5%)	
**S**	83/255 (32.5%)	38/112 (33.9%)	45/143 (31.5%)	
**Ceftriaxone (CRO)**				
**R**	255/255 (100.0%)	112/112 (100.0%)	143/143 (100.0%)	

***1* n/N (%)**

2 Pearson's Chi-squared test; Fisher's exact test

Table S2: Antibiotic susceptibility results of *K. pneumoniae* isolates per sampling area

**Characteristic**	**Total K. pneumoniae isolates*1***	**Sewage Location**		**p-value*2***
		**Blantyre**, N = 55*1*	**Soche**, N = 117*1*	
**Amoxicillin clavulanate (AUG)**				>0.9
**I**	55/172 (32.0%)	18/55 (32.7%)	37/117 (31.6%)	
**R**	94/172 (54.7%)	30/55 (54.5%)	64/117 (54.7%)	
**S**	23/172 (13.4%)	7/55 (12.7%)	16/117 (13.7%)	
**Meropenem (MEM)**				0.10
**I**	2/172 (1.2%)	1/55 (1.8%)	1/117 (0.9%)	
**R**	2/172 (1.2%)	2/55 (3.6%)	0/117 (0.0%)	
**S**	168/172 (97.7%)	52/55 (94.5%)	116/117 (99.1%)	
**Cotrimoxazole (SXT)**				0.7
**R**	167/172 (97.1%)	53/55 (96.4%)	114/117 (97.4%)	
**S**	5/172 (2.9%)	2/55 (3.6%)	3/117 (2.6%)	
**Doxycycline (DXT)**				>0.9
**I**	24/172 (14.0%)	7/55 (12.7%)	17/117 (14.5%)	
**R**	139/172 (80.8%)	45/55 (81.8%)	94/117 (80.3%)	
**S**	9/172 (5.2%)	3/55 (5.5%)	6/117 (5.1%)	
**Ciprofloxacin (CIP)**				0.3
**I**	37/172 (21.5%)	11/55 (20.0%)	26/117 (22.2%)	
**R**	121/172 (70.3%)	37/55 (67.3%)	84/117 (71.8%)	
**S**	14/172 (8.1%)	7/55 (12.7%)	7/117 (6.0%)	
**Gentamicin (GM)**				0.5
**R**	109/172 (63.4%)	37/55 (67.3%)	72/117 (61.5%)	
**S**	63/172 (36.6%)	18/55 (32.7%)	45/117 (38.5%)	
**Azithromycin (ATH)**				0.5
**R**	50/172 (29.1%)	18/55 (32.7%)	32/117 (27.4%)	
**S**	122/172 (70.9%)	37/55 (67.3%)	85/117 (72.6%)	
**Ceftriaxone (CRO)**				>0.9
**I**	3/172 (1.7%)	1/55 (1.8%)	2/117 (1.7%)	
**R**	169/172 (98.3%)	54/55 (98.2%)	115/117 (98.3%)	

1 **n/N (%)**

2 **Pearson's Chi-squared test; Fisher's exact test**

Table S3: Antibiotic susceptibility results of *E. cloacae* isolates per sampling area

**Characteristic**	**Total Enterobacter cloacae isolates**[1]	**Sewage Location**		**p-value**[2]
		**Blantyre**, N = 48[1]	**Soche**, N = 4[1]	
**Amoxicillin clavulanate (AUG)**				>0.9
**I**	4/52 (7.7%)	4/48 (8.3%)	0/4 (0.0%)	
**R**	47/52 (90.4%)	43/48 (89.6%)	4/4 (100.0%)	
**S**	1/52 (1.9%)	1/48 (2.1%)	0/4 (0.0%)	
**Meropenem (MEM)**				>0.9
**R**	2/52 (3.8%)	2/48 (4.2%)	0/4 (0.0%)	
**S**	50/52 (96.2%)	46/48 (95.8%)	4/4 (100.0%)	
**Cotrimoxazole (SXT)**				0.019
**I**	1/52 (1.9%)	1/48 (2.1%)	0/4 (0.0%)	
**R**	9/52 (17.3%)	6/48 (12.5%)	3/4 (75.0%)	
**S**	42/52 (80.8%)	41/48 (85.4%)	1/4 (25.0%)	
**Doxycycline (DXT)**				0.029
**I**	30/52 (57.7%)	30/48 (62.5%)	0/4 (0.0%)	
**R**	16/52 (30.8%)	13/48 (27.1%)	3/4 (75.0%)	
**S**	6/52 (11.5%)	5/48 (10.4%)	1/4 (25.0%)	
**Ciprofloxacin (CIP)**				0.10
**I**	4/52 (7.7%)	3/48 (6.3%)	1/4 (25.0%)	
**R**	12/52 (23.1%)	10/48 (20.8%)	2/4 (50.0%)	
**S**				

1 n/N (%)

2 Pearson's Chi-squared test; Fisher's exact test
